# COVID Vaccination in Cancer Patients: What Vaccination Priority Strategies Should There Be?

**DOI:** 10.3389/fonc.2021.641388

**Published:** 2021-02-04

**Authors:** Nicola Silvestris, Oronzo Brunetti, Renato Bernardini, Saverio Cinieri

**Affiliations:** ^1^ Medical Oncology Department, IRCCS IstitutoTumori “Giovanni Paolo II”, Bari, Italy; ^2^ Department of Biomedical Sciences and Human Oncology, University of Bari “Aldo Moro”, Bari, Italy; ^3^ Section on Pharmacology, Department of Biomedical and Biotechnological Sciences, University of Catania School of Medicine, Catania, Italy; ^4^ Medical Oncology & Breast Unit, Antonio Perrino Hospital, Brindisi, Italy

**Keywords:** COVID-19, SARS-CoV-2, vaccine, cancer patients, chemotherapy

## Introduction

At the height of the second wave of the COVID-19 pandemic, two fat lipid nanoparticle-vehicled RNA vaccines for SARS-COV-2 completed phase 3 trials with excellent results in terms of safety and efficacy (1, 2). In particular, the Pfizer vaccine trial considered 43,584 enrolled individuals with 170 COVID-19 positive subjects (162 and 8 in placebo and vaccine groups, respectively) (1). Similarly, the Moderna vaccine trial reported 90 and 5 infections (out of 30,000 volunteers enrolled) in placebo and vaccine groups, respectively. Both vaccines achieved 95% efficacy (2). Fever, fatigue, and headache were the most common side effects with higher incidence for Moderna vs the Pfizer vaccine (1, 2).

## Background and Rationale

Considering the urgent need to define priorities in selecting the subgroups of the population requiring such a vaccination, national vaccination plans have been elaborated. In particular, the Italian national strategic plan lists the following priority categories: i) doctors and health professionals; ii) residents and staff of residential care centers for the elderly; iii) elderly population (at first those aged over 80 years, and then between 60 and 79 years); and iv) population with at least one chronic comorbidity (3). According to these indications, it is estimated that 27 million Italian people (20 million belonging to the first three categories and 7 million with chronic diseases) will require early vaccination (3). Considering that two doses are scheduled for each patient, it will be necessary, in the beginning, to define who should be vaccinated among individuals with chronic diseases. Oncologic patients represent one of the most fragile categories (4). In this line, a retrospective study conducted at the Memorial Sloan Kettering Cancer Center showed that hematologic malignancies are associated with increased COVID-19 severity (HR 1.90; 95% CI: 1.30-2.80), and lung cancer patients experienced a higher rate of serious COVID-19 symptoms (HR 2.0; 95% CI: 1.20 - 3.30) (5). The rate of adverse events was lower in a time-matched population of patients with cancer without COVID-19.

According to AIRTUM data, about 6% of the Italian population is affected by a neoplastic disease, corresponding to over 3.5 million people (6). Keeping these data in mind, the key question is: *what vaccination priority strategies in cancer patients should there be?*

## Discussion

It is well known that cancer patients do not represent a homogeneous population according to the four categories under which cancer treatments fall proposed by Schrag et al. (curative potential, moderate clinical importance, marginal impact on quality or quantity of life, and survivorship and surveillance) (7). According to these categories and the available therapeutic strategies, we believe that the following subsets of cancer patients should be vaccinated first:

- *subjects undergoing radical surgery who are candidates for adjuvant systemic treatments* starting within 1-2 months after surgery aimed to improve both relapse-free survival and overall survival (8); in these cases, the first dose of the vaccine should be administered within 7-10 days from surgery and the second after another 3 weeks, taking into account the timing of the adjuvant treatments. A similar approach is considered in the management of cancer patients who underwent splenectomy requiring pneumococcal and meningococcal vaccinations before the beginning of systemic therapies (9);
- *patients with advanced disease* (especially lung cancer patients) whose tumor burden and biological assessment allows for a one-month postponement of the beginning of systemic treatments.

Recently, ESMO published 10 statements to address these issues and concerns (9). However, several questions still remain open for the other subset of cancer patients: *which criteria could be applied for advanced patients during systemic treatments, including chemotherapy, targeted therapies, and immunotherapy? What could the management be for patients under hormonal therapies? Are cancer patients previously infected by SARS-CoV2 candidates to receive the vaccine? What kind of interactions could occur between antiviral vaccines and both antineoplastic therapies and the associated ancillary drugs including steroids? How long should immunity be expected to last in these patients?*

Today, national health institutions and the scientific community are urged to answer these questions. To fulfill this commitment, in the absence of published data, large-scale collaborative post-trial, and registry monitoring studies are required.

## Author Contributions

Conception or design of the work: NS. Literature research: OB. Drafting and writing the article: OB, NS. Critical revision of the article: RB, SC. All authors contributed to the article and approved the submitted version.

## Conflict of Interest

The authors declare that the research was conducted in the absence of any commercial or financial relationships that could be construed as a potential conflict of interest.
